# The use of cladribine tablets in patients with MS beyond the age of 50: experiences from a large tertiary German centre

**DOI:** 10.1186/s42466-026-00502-7

**Published:** 2026-06-02

**Authors:** Franziska Axhausen, Stephanie Wolff, Romy Baumgart, Borros Arneth, Anne Mrochen, Heidrun H. Kraemer, Steffen Pfeuffer

**Affiliations:** 1 Department of Neurology, Justus-Liebig-University Giessen, Giessen, Germany; 2 Institute of Laboratory Medicine and Pathobiochemistry, Justus-Liebig-University Giessen, Giessen, Germany; 3 Department of Neurology, University Hospital Giessen and Marburg, Justus-Liebig-University Giessen, Klinikstr. 33, 35392 Giessen, Germany

**Keywords:** Multiple sclerosis, Cladribine, Older patients

## Abstract

**Background:**

Evidence for disease-modifying therapies in patients with multiple sclerosis (MS) aged over 50 years is limited because this population is often excluded from randomized trials. With aging, MS increasingly reflects progression independent of relapse activity (PIRA), while adverse event risk increases. Cladribine is an immune reconstitution therapy with established efficacy in younger populations, but real-world data in older patients, particularly after prior high-efficacy therapies, are scarce.

**Methods:**

We conducted a prospective observational study of 95 patients aged over 50 years with active MS treated with cladribine at a tertiary MS center in Giessen, Germany. Patients were stratified by prior therapy (treatment-naïve, platform therapy, high-efficacy therapy). Outcomes included relapses, MRI activity, confirmed disability worsening (CDW), confirmed disability improvement (CDI), no evidence of disease activity (NEDA-3/4), lymphocyte dynamics, adverse events, and serum neurofilament light chain (NfL).

**Results:**

Mean age was 55.9 ± 4.1 years, baseline EDSS was 2.5 ± 1.0. Total follow-up duration was 137 patient-years. Annualized relapse rate decreased from 0.66 before treatment to 0.044 during follow-up. Six relapses and six new T2 lesions (each 6.3%) occurred during follow-up. CDW was observed in 10 patients (11.8%), while 21 patients (24.7%) showed sustained CDI. NEDA-3 was achieved by 66 patients (77.6%) and NEDA-4 by 65 patients (76.5%). No clear differences between prior-treatment groups were observed. Serum NfL levels decreased during treatment. Lymphopenia was predominantly grade I–II. Infections were infrequent (27.4%) and mostly mild.

**Conclusions:**

Cladribine showed sustained effectiveness and an acceptable safety profile in MS patients over 50 years.

## Introduction

The prevalence of multiple sclerosis (MS) in patients over the age of 50 has increased significantly in recent years, partly due to improved therapeutic options and rising life expectancy [1]. However, evidence on the treatment of this age group remains limited [2], particularly as individuals over the age of 50 are usually excluded from randomized controlled trials [1].

In addition, the disease course of MS evolves with advancing age and disease duration, shifting from a predominantly inflammatory phenotype to one characterized more by progression independent of relapse activity (PIRA) [3, 4]. On the other hand, the risk of adverse events increases with age, due to enhanced susceptibility to infections associated with immunosenescence [5, 6], higher burden of comorbidities and higher risk of polypharmacy [1].

Among currently approved disease-modifying therapies (DMTs), cladribine induces a selective depletion of lymphocytes followed by immune reconstitution offering “drug-free remission” [7]. Its efficacy and safety were first demonstrated in the CLARITY trial in 2010, and it has been approved for use in Europe since 2017 for adult patients [7].

While its overall safety and efficacy have been confirmed in various real-world settings [8–10], these findings predominantly pertain to patients under the age of 50. Older patients with a longer disease duration often have prior exposure to other DMTs; however, data on the effectiveness and safety of cladribine — particularly following a switch from high-efficacy treatments such as B-cell-depleting therapies (BCT) — are still scarce [11].

To address these knowledge gaps, we present real-world data on the use of cladribine in patients over the age of 50 from our large tertiary care center in Giessen, Germany.

## Methods

### Patients

Patients scheduled to receive cladribine for treatment of active, relapsing MS according to the 2017 revised McDonald criteria [12] were enrolled in our local prospective registry. Active MS was defined as either ongoing clinical activity (relapses, disability worsening, new T2-hyperintense MRI lesions) or the use of high-efficacy therapies (sphingosine-1-phosphate receptor modulators (S1PRM), BCT, natalizumab) before switching to cladribine. None of the patients fulfilled the criteria of secondary progressive multiple sclerosis [13]. The treatment decision was made independently of registry inclusion, based on national and international guidelines, and product characteristics [14]. Before treatment initiation, we ensured that all patients received herpes zoster vaccination and documented vaccination status in our electronic health record system.

Following treatment, our patients were evaluated at least semiannually including the evaluation of clinical relapses, assessment of the Expanded Disability Status Scale (EDSS) scores as well as MRI parameters. MRI scans were conducted semiannually as well and were evaluated for development of new T2-hyperintense lesions at our center.

Prospectively collected parameters also included blood leukocyte and lymphocyte counts as well as serum immunoglobulin G (IgG) levels. Among patients who had previously received BCT, peripheral CD19 + B-cell counts were assessed additionally. Furthermore, we actively interviewed patients for development of upper respiratory tract infections, urinary tract infections and herpes zoster.

Following introduction into clinical routine in December 2024, all patients underwent evaluation of neurofilament light chain (NfL) serostatus. NfL levels were assessed using the commercially available assay provided by Siemens Healthineers. All analyses were conducted in our local accredited central laboratory during clinical routine.

For the current study, all patients above the age of 50 years were included. Patients with comorbid autoimmunity or other significant comorbidities (neoplastic disease, other neurologic or psychiatric condition) were excluded.

### Treatment outcomes

We separated our cohort into three groups according to their prior DMT. Naïve patients were termed “1st line”; patients who were treated with platform therapies (glatiramer acetate, teriflunomide, dimethyl fumarate) before were included in the group “2nd line”, “3rd line” group included patients who were treated with high-efficacy therapies (S1PRM, BCT, natalizumab) before.

Outcome parameters were the number of relapses, confirmed disability worsening (CDW), relapse-associated worsening (RAW), progression independent of relapse activity (PIRA), and confirmed and sustained disability improvement (CDI). CDW was defined as an EDSS increase of ≥ 1.0 point for baseline EDSS 0–5.5 or ≥ 0.5 points for baseline EDSS ≥ 6.0, confirmed at a subsequent follow-up visit within 6 months. RAW was defined as CDW occurring in association with a documented relapse within the preceding 6 months. PIRA was defined as CDW without a documented relapse within the preceding 6 months. CDI was defined as an EDSS decrease of ≥ 1.0 point for baseline EDSS 0–5.5 or ≥ 0.5 points for baseline EDSS ≥ 6.0, confirmed at a subsequent follow-up visit within 6 months.

Combined outcome parameters were termed as no evidence of disease activity 3 (NEDA-3), defined as absence of relapses, new T2-hyperintense MRI lesions (T2L), and CDW, and no evidence of disease activity 4 (NEDA-4), which is defined by the criteria of NEDA-3 and the absence of NfL levels exceeding the age-specific cut-off among patients with available NfL data.

Outcome parameters were assessed in patients who had a follow-up longer than 6 months.

### Statistical analysis

We performed descriptive statistics, including the calculation of annualized relapse rate (ARR), annualized progression rate (APR) and annualized event rate (AER).

For evaluation of effectiveness, we generated Kaplan-Meier plots using relapses, occurrence of new T2L and CDW as outcome measures. Patients without an event were censored at the last follow-up visit.

NfL levels were additionally analyzed using a linear mixed-effects model [15].

Differences between treatment groups were assessed using Cox regression, adjusted for age, sex and disease duration. The “1st line” group served as the reference group in the Cox regression analyses, resulting in the following comparisons: “2nd vs 1st line” and “3rd vs 1st line”. *P* < 0.05 was considered significant. All analyses were performed using SPSS (version 29.0.2.0).

## Results

### Baseline demographics

Between November 2020 and September 2025, 104 patients were treated with cladribine at our center. Of those, 98 patients were older than 50 years. After excluding three patients with significant comorbidities (all three had additional autoimmune diseases), 95 patients were included in this analysis.

The overall cohort had a mean age of 55.9 ± 4.1 years (ranging from 50 to 66 years) and a mean disease duration of 74 ± 66 months. The cohort comprised 41 patients aged 50–54 years (43.2%), 33 patients aged 55–59 years (34.7%), and 21 patients aged 60–66 years (22.1%). The total follow-up duration of the cohort was 137 patient-years, with a median follow-up duration of 18 months (range 3–47 months).

Disease burden at baseline, assessed by mean EDSS, annualized relapse rate (ARR), and confirmed disability worsening (CDW), did not differ between the three age groups (mean ARR: 50–54 years, 0.66; 55–59 years, 0.67; 60–66 years, 0.67; mean EDSS: 50–54 years, 2.6; 55–59 years, 2.5; 60–66 years, 2.5; mean CDW: 50–54 years, 0.17; 55–59 years, 0.25; 60–66 years, 0.17).

The mean Expanded Disability Status Scale (EDSS) score prior to initiation of cladribine treatment was 2.5 ± 1.0 across the entire cohort. Baseline demographic and clinical characteristics of the three treatment-line groups are presented in Table 1. No significant differences in age were observed between the groups. Compared with the other groups, patients treated with cladribine as ”1st line” therapy had the lowest disease burden, reflected by the lowest mean EDSS score (1.8 ± 0.8) and the lowest mean MRI lesion load (12.6 ± 3.9). In contrast, the “3rd line” treatment group exhibited the highest disease burden but the lowest disease activity, with a baseline annualized relapse rate (ARR) of 0.3.

**Table 1:** Baseline demographics

	1st line (*n* = 33)	2nd line (*n* = 25)	3rd line (*n* = 37)
Female sex, *n* (%)	17 (52)	17 (68)	23 (62)
Age, years, mean (SD)	56.7 (4.5)	56.2 (4.4)	54.9 (3.4)
MS duration, months, mean (SD)	8.9 (14.9)	99.8 (54.8)	114.8 (56.2)
Baseline EDSS, mean (SD)	1.8 (0.8)	2.5 (0.8)	3.2 (1.0)
Baseline T2 lesion load, mean (SD)	12.6 (3.9)	20.9 (9.3)	26.7 (11.6)
Annualized relapse rate, mean (SD)	1.1 (0.3)	0.6 (0.6)	0.3 (0.5)
CDW within past 12 months, n (%)			
None	0	20 (80)	30 (81)
RAW	33 (100)	2 (8)	0
PIRA	0	3 (12)	7 (19)
Last previous DMT, n (%)			
Naïve	33 (100)	0	0
Injectable	0	13 (52)	0
Teriflunomide	0	8 (32)	0
‘Dimethyl fumarate	0	4 (16)	0
S1P-Receptormodulator	0	0	15 (40)
Natalizumab	0	0	8 (22)
BCT	0	0	14 (38)

SD = standard deviation; MS = multiple sclerosis; EDSS = Expanded Disability Status Scale; CDW = confirmed disability worsening; RAW = relapse associated worsening; PIRA = progression independent of relapsing activity; DMT = disease modifying therapy; BCT = B-cell-depleting therapy

This apparent stability may be explained by the fact that most patients in the “3rd line” group switched therapy due to adverse events (23 patients, 62%) or JC virus seroconversion (8 patients, 22%), rather than due to ongoing disease activity (6 patients, 16%). In contrast, patients in the “2nd line” group switched to cladribine because of disease activity. The mean washout period was 15.8 ± 14.6 days in the “2nd line” and 91.4 ± 79.3 days in the “3rd line” group. Only one patient in the “3rd line” group experienced a relapse during the washout period.

### Effectiveness outcomes

Effectiveness outcomes were analyzed in 85 patients with a minimum follow-up duration of 6 months. Of these, 33 patients belonged to the “1st line” group, 23 to the “2nd line” group, and 29 to the “3rd line” group.

**Fig. 1:**
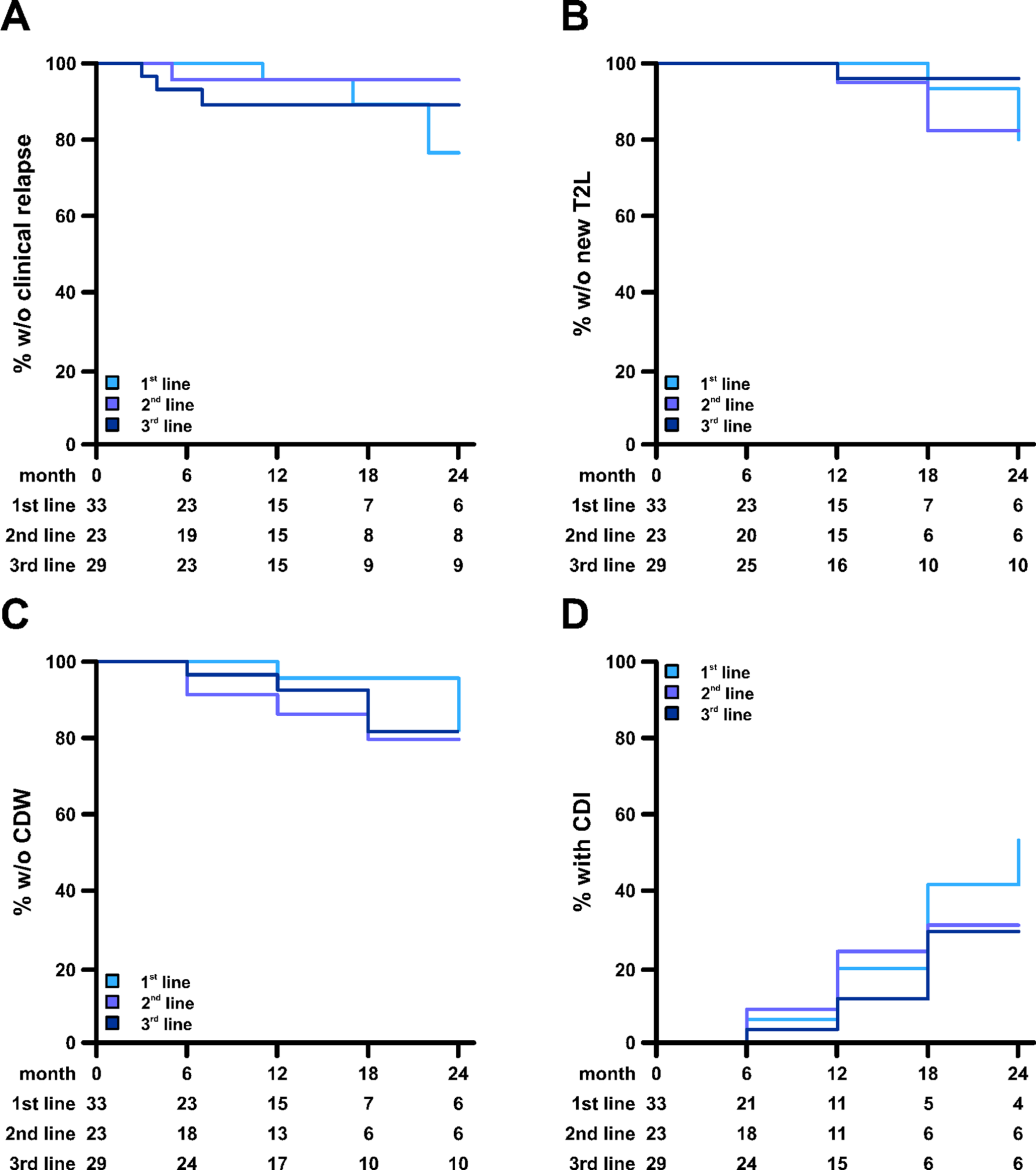
Effectiveness outcomes.** A**: Kaplan-Meier plots for time to first relapse among “1st line”, “2nd line” and “3rd line” patients. Numbers at risk are indicated below the plot. **B**: Kaplan-Meier plots for time to first new T2 hyperintense MRI lesion (T2L) among “1st line”, “2nd line” and “3rd line” patients. Numbers at risk are indicated below the plot. **C**: Kaplan-Meier plots for time to first confirmed disability worsening (CDW) among “1st line”, “2nd line” and “3rd line” patients. Numbers at risk are shown below the plot. **D**: Kaplan-Meier plots for time to first confirmed and sustained disability improvement (CDI) among “1st line”, “2nd line” and “3rd line” patients. Numbers at risk are indicated below the plot

**Fig. 2:**
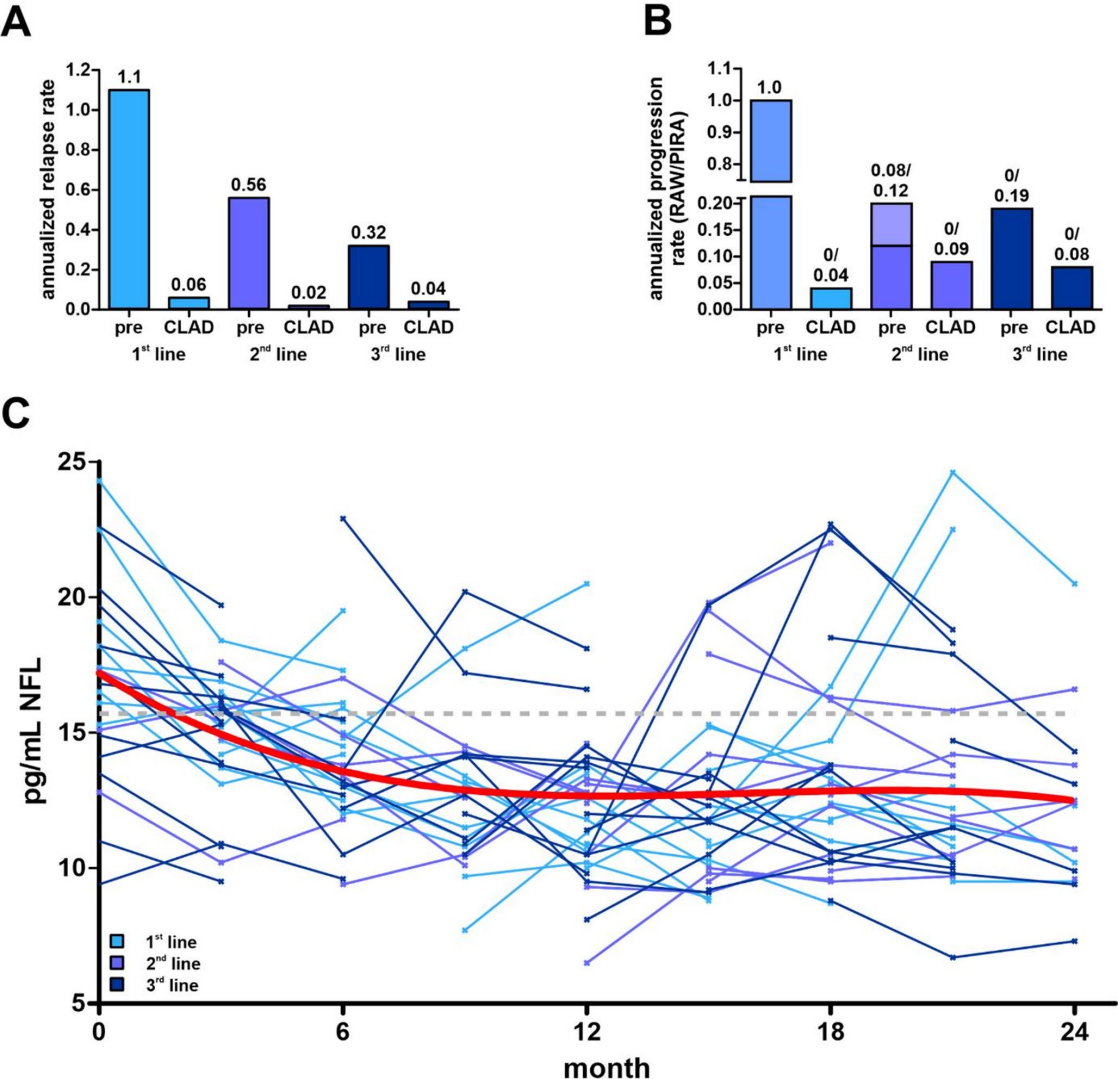
Effectiveness outcomes 2.** A**: Annualized relapse rates (ARR) before (pre) and during treatment with cladribine (CLAD) among “1st line”, “2nd line” and “3rd line” patients. ARR are indicated above the graphs. **B**: Annualized progression rate (APR), separated in relapse associated worsening (RAW) and progression independent of relapsing activity (PIRA). Rates are indicated above the graphs (RAW/ PIRA). **C**: Serum NfL levels (pg/mL) of each patient during treatment with cladribine subordinate to the month of measurement. Values of one patient are connected, affiliation to the groups “1st line”, “2nd line” and “3rd line” is indicated by color. A trend line was fitted using a linear mixed-effects model (LMM) (red line)

Overall, six relapses were observed during follow-up: three in the “1st line” group (9.1%), one in the “2nd line“ group (4.3%), and two in the “3rd line” group (6.9%) (HR 0.66, 95% CI: 0.002–1.766 p = 0.105; 0.79, 95% CI: 0.002–2.779 p = 0.163) **(**Fig. 1A**)**. Prior to cladribine treatment, the annualized relapse rate (ARR) for the overall cohort was 0.66. Following treatment initiation, the ARR decreased to 0.044. The most pronounced reduction was observed in treatment-naïve patients **(**Fig. 2A**).**

The occurrence of new T2-hyperintense lesions (T2L) was low across all groups, with six patients (6.3%) showing MRI progression during follow-up. Of these, two patients (6.1%) were in the “1st line” group, three in the “2nd line” group (13.0%), and one in the “3rd line” group (3.4%) (HR 2.63, 95% CI: 0.192–37.542, *p* = 0.463; HR 0.54, 95% CI: 0.027–10.629; *p* = 0.685) **(**Fig. 1B**)**.

Across the effectiveness cohort, 10 patients (11.8%) experienced confirmed disability worsening (CDW) during follow-up, representing a reduction compared with baseline, where CDW had been observed in 45 of 95 patients (47.4%). No significant differences were observed between the treatment-line groups (HR 2.56, 95% CI: 0.297–22.15, *p* = 0.392; HR 2.41, 95% CI: 0.222–26.189; *p* = 0.469) **(**Fig. 1C**).**

To further characterize disability progression, annualized progression rates were analyzed by distinguishing between relapse-associated worsening (RAW) and PIRA. Prior to cladribine treatment, the highest annualized progression rate was observed in treatment-naïve patients and was exclusively relapse-associated. In the other groups, progression rates before treatment were low and predominantly relapse-independent (“2nd line” group: RAW 0.08, PIRA 0.12; “3rd line” group: PIRA 0.19). Following initiation of cladribine, progression rates decreased in all groups and were exclusively relapse-independent **(**Fig. 2B**).**

66 of 85 patients (77.6%) achieved no evidence of disease activity (NEDA-3; defined as absence of relapses, new T2L, and CDW) during follow-up. The “1st line” group showed a slightly higher proportion of NEDA-3 status (28 patients, 84.8%) compared with the “2nd line” (17 patients, 73.9%) and “3rd line” groups (21 patients, 72.4%).

Notably, confirmed and sustained disability improvement (CDI) was observed in 21 patients (24.7%), predominantly in the youngest age group (12 patients aged 50–54 years). CDI occurred across all treatment-line groups, including 9 patients (27.3%) in the “1st line” group, 6 patients (26.1%) in the “2nd line” group, and 6 patients (20.7%) in the “3rd line” group (HR 1.26, 95% CI: 0.332–4.753 *p* = 0.737; 0.91, 95% CI: 0.211–3.949 *p* = 0.903) **(**Fig. 1D**)**.

A total of 257 serum NfL measurements were obtained during follow-up from 86 patients at different time points (mean of 2.98 measurements per patient). At baseline mean serum NfL in 22 patients was 16.9 ± 3.6 pg/mL, with no values exceeding the age-adjusted cut-off. After 24 months of cladribine treatment, mean NfL decreased to 12.1 ± 3.1 pg/mL (measured in 16 patients). Among patients with available NfL data and at least 6 months of follow-up, 65 of 85 patients (76.5%) achieved NEDA-4 status, defined as NEDA-3 and NfL levels below the age-specific cut-off. A reduction in serum NfL during treatment was observed in 51 of 86 patients (59.3%). In addition, a trend toward decreasing serum NfL levels over treatment duration was observed **(**Fig. 2C**)**.

During follow-up, serum NfL levels above the age-adjusted cut-off were detected in nine patients. Eight of these patients experienced CDW during the observation period. In all cases, elevated NfL levels were documented either concurrently with CDW or within the preceding three months. No comparable association was observed for relapses or MRI progression. These findings suggest that serum NfL, even during cladribine treatment, may serve as a useful biomarker for monitoring disease progression, particularly with respect to disability worsening.

### Safety outcomes

**Fig. 3:**
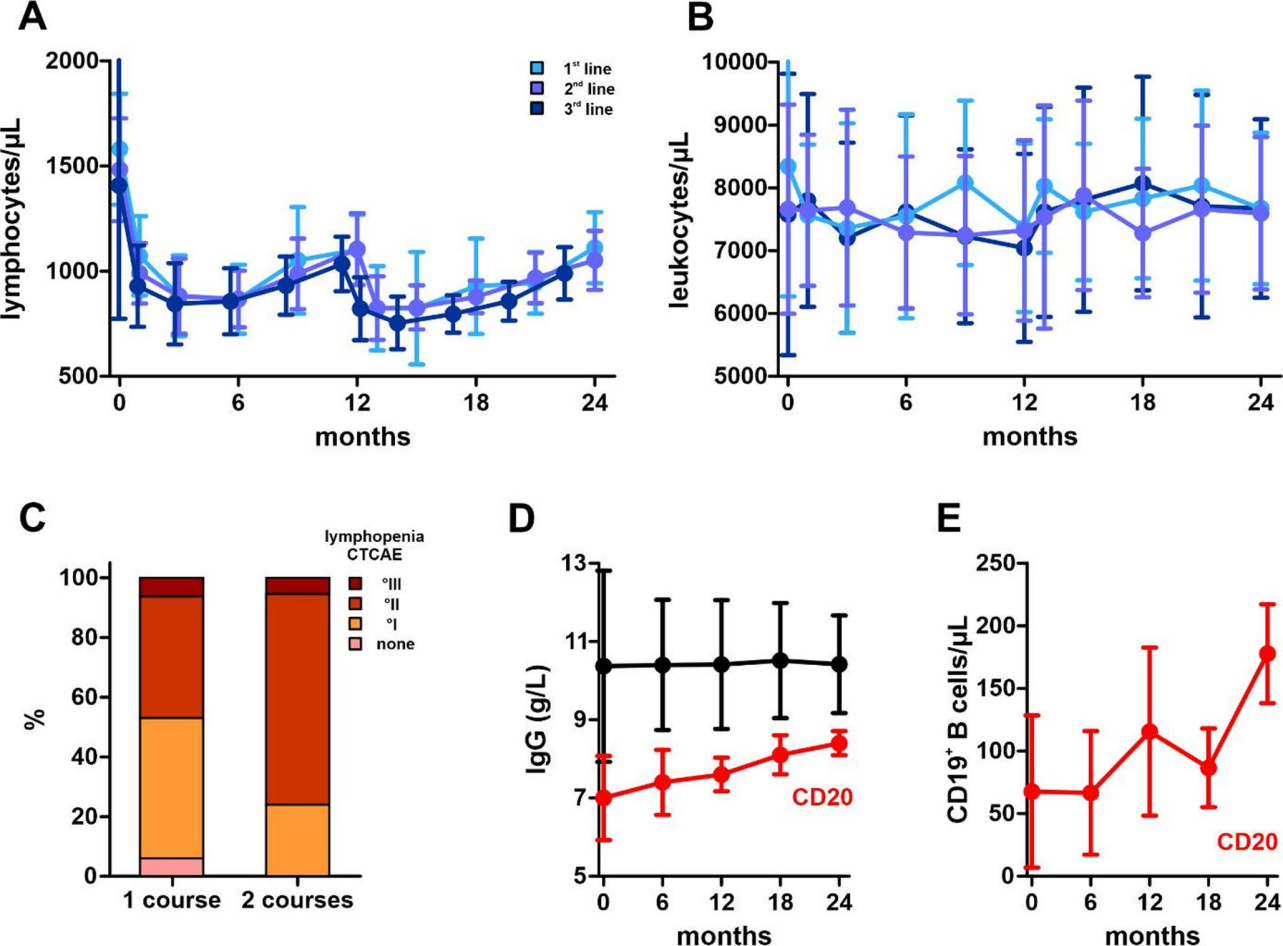
Leukocyte levels and IgG during treatment with cladribine. **A**: Mean blood lymphocyte counts (/µl) and standard deviations during treatment with cladribine among “1st line”, “2nd line” and “3rd line” patients. **B**: Mean blood leukocyte counts (/µl) and standard deviations during treatment with cladribine among “1st line”, “2nd line” and “3rd line” patients. **C**: Relative frequence of lymphopenia at the nadir, classified according to the CTCAE in % of all patients, after the first (year 1) and second (year 2) treatment course of cladribine. **D**: Serum IgG levels (g/L) of the whole cohort (black) and of patients treated with B-cell depleting therapy before (CD20, red). **E**: CD19 + B-cell levels (blood, /µL), during treatment with cladribine of patients treated with B-cell-depleting therapy before

Baseline mean lymphocyte counts were also within the normal range in the overall cohort (1486.9 ± 451/µL) and across all treatment-line groups (“1st line“: 1579.7 ± 263.8/µL; “2nd line“: 1482.4 ± 244.5/µL; “3rd line”: 1407.3 ± 633.3/µL) (Fig. 3A). Baseline lymphopenia was observed in 10 patients, all of whom had received prior DMT: one patient belonged to the ”2nd line“ group and nine patients to the “3rd line” group.

Following cladribine administration, the expected pattern of lymphocyte decline to a nadir followed by gradual recovery was observed. Lymphocyte counts at nadir were classified according to the Common Terminology Criteria for Adverse Events (CTCAE) [7] **(**Fig. 3C**).** Overall, lymphopenia was predominantly mild to moderate, with 30 patients (31.6%) experiencing grade I and 58 patients (61.1%) grade II lymphopenia. Grade III lymphopenia occurred in five patients (5.3%), while grade IV lymphopenia was not observed.

Higher-grade lymphopenia was not associated with age or treatment line. In the “1st line“ group, 12 patients developed grade I lymphopenia, 19 grade II, and three grade III. In the “2nd line“ group, 10 patients experienced grade I and 15 grade II lymphopenia. In the “3rd line” group, two patients had no lymphopenia, eight developed grade I, 24 grade II, and three grade III lymphopenia.

Mean leukocyte counts were within the normal range at baseline in the overall cohort (7680.5 ± 2122.8/µL) as well as in all treatment-line groups (“1st line“: 8342.4 ± 2069.7/µL; “2nd line“: 6961.2 ± 1663.6/µL; “3rd line”: 7576.2 ± 2237.6/µL) and remained stable throughout cladribine treatment **(**Fig. 3B**).**

Mean baseline IgG levels were 10.4 ± 2.3 g/L over the entire cohort. In patients without prior BCT, mean baseline IgG levels were 11.0 ± 1.8 g/L and remained stable during cladribine treatment. In contrast, patients previously treated with BCT showed reduced baseline CD19⁺ B-cell counts and IgG levels (6.8 ± 5.8/µL and 7.0 ± 1.1 g/L). During cladribine treatment, both CD19⁺ B-cell counts and IgG levels gradually recovered, reaching 11.8 ± 3.1/µL and 8.4 ± 0.4 g/L at month 24.

### Adverse events

**Fig. 4:**
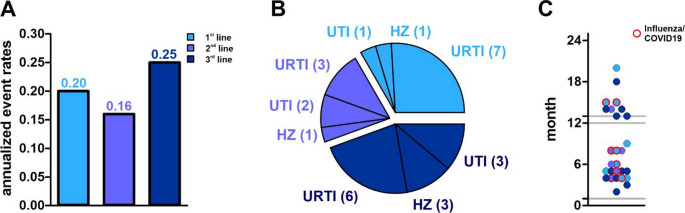
Adverse events **A**: Annualized event rates (AER) of adverse events during treatment with cladribine among “1st line”, “2nd line” and “3rd line” patients. AER are indicated above the graphs. **B**: Absolute frequency of infections among “1st line”, “2nd line” and “3rd line” patients. URTI: upper respiratory tract infections. UTI: urinary tract infections. HZ: herpes zoster. **C**: Time to infection for each patient among “1st line”, “2nd line” and “3rd line” patients. Influenza virus and coronavirus disease 19 (COVID19) infections are indicated red

During follow-up, no novel safety signals were identified. Overall, 26 patients (27.4%) experienced at least one adverse event, corresponding to an annualized event rate of 0.19. The risk of experiencing an adverse event did not differ significantly between treatment groups (HR 2.41, 95% CI 0.65–8.98 *p* = 0.191; HR 2.31, 95%-CI 0.57–1.00; *p* = 0.239) or age (HR 1.76, 95% CI 0.64–4.82 *p* = 0.273; HR 1.22, 95%-CI 0.33–4.54; *p* = 0.772) **(**Fig. 4A**).**

Reported adverse events predominantly comprised infections, including respiratory tract infections, urinary tract infections, and herpes zoster. The most frequent were respiratory tract infections, with 16 events in total (16.8%), including three cases of COVID-19 pneumonia, one case of influenza, four cases of pneumonia, and seven other upper respiratory tract infections. Less frequent infections included urinary tract infections (6 events, 6.3%) and herpes zoster (5 events, 5.2%) (Fig. 4B). All infections resolved during the follow-up period. No hospitalizations, treatment discontinuations, or malignancies occurred during follow-up.

Most infections occurred 4, 5, 13, and 14 months after treatment initiation (Fig. 4C), coinciding with the lymphocyte nadir. Higher-grade lymphopenia was associated with an increased incidence of infections: infections occurred in 6 patients (20%) with grade I lymphopenia, 15 patients (26%) with grade II lymphopenia, and in all patients (100%) with grade III lymphopenia.

## Discussion

Previous studies have shown that even older patients can benefit from disease-modifying therapies (DMTs), including high-efficacy therapies (HET), due to a reduction in relapse risk. Also, in patients over the age of 50, relapses have been associated with increased disease activity and, over time, with PIRA [8]. It has also been demonstrated that therapeutic escalation can lead to improved disease stability and therefore quality of life in this patient group [8].

However, the effectiveness of HET appears to decline with increasing age. Nevertheless, long-term data suggest that HET can still reduce disease progression in older patients, indicating that such therapies may remain beneficial in this population [16], although the risk of adverse events increases with age [1].

In this study, we present results from our prospective real-world experience treating 95 patients over the age of 50 with cladribine.

We observed satisfactory effectiveness of cladribine across the cohort, regardless of prior DMT exposure. This was reflected in low inflammatory disease activity during treatment, indicated by a low rate of new relapses and newly emerging T2-hyperintense MRI lesions. Additionally, the rate of confirmed disability worsening (CDW) was low across all patient groups after initiating cladribine and most of our patients reached NEDA-3 status.

These findings are consistent with earlier reports [17–19]. Most recently, an Italian study demonstrated reductions in relapses and PIRA in patients both younger and older than 50 years [20].

Similarly, as reported by Manni et al. [21], we observed a treatment- and time-dependent reduction of serum NfL levels during treatment with cladribine.

Notably, a substantial proportion of our patients experienced confirmed and sustained disability improvement. This stands in contrast to recently published data suggesting that, although DMTs may reduce inflammatory activity (e.g., relapses, MRI lesions) in patients with late-onset MS, they do not affect disability worsening or conversion to secondary progressive MS [22]. One hypothesis is that cladribine may exert central nervous system effects due to blood-brain barrier penetration [9]. However, this hypothesis remains speculative and requires further investigation.

Furthermore, we did not observe clear differences in the effectiveness of cladribine in patients with different prior therapies. This is contrary to previous assumptions [6, 8]. However, in our study, treatment groups differed at baseline and event numbers were low, which precludes firm comparative conclusions. Patients who had previously been treated with highly effective therapies such as BCT or natalizumab demonstrated stable disease courses following the switch to cladribine in our cohort. Similarly, the phase 4 CLADRINA study reported low relapse rates after switching from natalizumab to cladribine [23].

During follow-up, we observed mild lymphopenia, consistent with previous reports [7]. Grade III lymphopenia occurred infrequently, and no cases of grade IV lymphopenia were observed. In contrast to previous findings from our group indicating a higher risk of lymphopenia in patients previously treated with fingolimod [8], this association was not confirmed in the current study.

Consistent with our results, Giovannoni et al. reported a similar risk of lymphopenia in patients both above and below the age of 50. However, they noted an increased infection risk among older patients with lymphopenia [24].

In our cohort, the overall infection rate was low. Herpes zoster infections were less frequent than previously reported [8]. This may be attributed to strict adherence to local vaccination guidelines prior to initiating cladribine therapy. However, we also observed a temporal correlation between the nadir and the time point when the most infections occurred. Overall infection rate was slightly higher than reported in younger patients [10].

Our study is characterized by its relatively large sample size and the inclusion of patients with a wide range of prior therapies. A particularly noteworthy finding is the unusually high proportion of patients who experienced not only disease stability but improvement of disability during treatment. The monitoring of NfL levels also served as an additional biomarker of disease activity.

However, several limitations related to the study design must be acknowledged. This was an observational study without a control group and small treatment-related subgroups. Furthermore, follow-up durations varied between patients, and in some cases were shorter than 24 months. For these reasons, the findings of our study are rather hypothesis-generating. Extending the follow-up period would be valuable to assess long-term therapeutic outcomes. In addition, establishing a propensity-matched control group of older patients who were not switched to cladribine would be helpful for comparative analysis. Because NfL measurements were introduced into routine diagnostics only in December 2024, baseline data exist only in a subset of our patients, and the number of measurements differs. Thus, NfL data must be interpreted with caution, and more standardized data are needed to draw definitive conclusions.

In summary, our real-world experience treating 95 patients over the age of 50 with cladribine adds to the existing body of evidence. Our findings suggest that cladribine is a safe treatment option for older patients with MS, associated with a low rate of infections. Furthermore, in our cohort, we observed reduced inflammatory activity, minimal disability progression, and — in a notable proportion of patients — even sustained improvement of disability during treatment with cladribine.

## Data Availability

Anonymized patient data will be shared upon reasonable requests from qualified investigators.

## Abbreviations

AER: Annualized event rate
APR: Annualized progression rate
ARR: Annualized relapse rate
BCT: B–cell–depleting therapy
CDI: Confirmed disability improvement
CDW: Confirmed disability worsening
CLARITY: Cladribine tablets treating multiple sclerosis orally (clinical trial name)
CTCAE: Common terminology criteria for adverse events
DMT(s): Disease–modifying therapy(ies)
EDSS: Expanded disability status scale
HET: High–efficacy therapy
IgG: Immunoglobulin G
MRI: Magnetic resonance imaging
MS: Multiple sclerosis
NEDA: 3–No evidence of disease activity–3
NEDA: 4–No evidence of disease activity–4
NfL: Neurofilament light chain
PIRA: Progression independent of relapse activity
RAW: Relapse–associated worsening
S1PRM: Sphingosine–1–phosphate receptor modulator
SPSS: Statistical package for the social sciences
T2L: T2–hyperintense lesions
